# Real-World Management and Outcomes of Probable and Indeterminate UIP Patterns on HRCT: A Retrospective Cohort Study and Implications for the 2022 IPF Guideline

**DOI:** 10.3390/jcm15207786

**Published:** 2026-10-09

**Authors:** Gulce Cansu Serin, Ebru Dumlupınar, Caglar Uzun, Ozlem Ozdemir Kumbasar

**Affiliations:** 1 Department of Chest Diseases, Ankara University Faculty of Medicine, Cebeci, 06590 Ankara, Turkey; ozlemozdemir@yahoo.com; 2 Department of Biostatistics, Ankara University Faculty of Medicine, Cebeci, 06590 Ankara, Turkey; 3 Department of Radiology, Ankara University Faculty of Medicine, Cebeci, 06590 Ankara, Turkey

**Keywords:** idiopathic pulmonary fibrosis, interstitial lung disease, idiopathic interstitial pneumonia, probable usual interstitial pneumonia, interstitial fibrosis, surgical lung biopsy

## Abstract

**Background:** The 2018 ATS/ERS/JRS/ALAT guideline conditionally recommended surgical lung biopsy (SLB) for patients with probable or indeterminate usual interstitial pneumonia (UIP) patterns on high-resolution computed tomography (HRCT). This study assessed the real-world use of this recommendation and outcomes of patients with probable or indeterminate UIP. **Methods:** HRCT scans of 1255 patients with interstitial lung disease (ILD) followed between 2012–2021 were retrospectively reviewed. A total of 185 patients met the inclusion criteria. We re-evaluated the HRCT scans and classified them through multidisciplinary discussion (MDD) involving one thoracic radiologist, one pulmonary pathologist, one rheumatologist, and at least three pulmonologists specializing in ILD. We analyzed clinical features, biopsy rates, final diagnoses, and survival outcomes. **Results:** SLB was performed in 10.8% of patients and met the predefined criteria for clinical biopsy success in 50%. Among patients with probable UIP who were ultimately diagnosed with IPF, 32 of 38 (84.2%) received the diagnosis without SLB. The remaining six (15.8%) underwent SLB, of whom five met predefined criteria for clinical biopsy success. Final diagnoses included IPF (23.8%), hypersensitivity pneumonitis (16.8%), and connective tissue disease-associated ILD (16.2%). Crude mortality proportions did not differ significantly between the probable and indeterminate UIP groups (*p* = 0.173). However, time-to-event analyses demonstrated shorter survival in the probable UIP group (log-rank *p* = 0.019), and probable UIP was associated with a higher mortality hazard after adjustment for age, sex, and smoking history (adjusted HR 1.91, 95% CI 1.06–3.42; *p* = 0.030). This association was attenuated and no longer statistically significant after additional adjustment for cardiovascular disease (adjusted HR 1.71,95% CI 0.93–3.14; *p* = 0.082). **Conclusions:** These findings support an individualized, multidisciplinary diagnostic approach and are consistent with the diagnostic flexibility introduced in the 2022 IPF guideline.

## 1. Introduction

Idiopathic pulmonary fibrosis (IPF) is a chronic, progressive, and fibrotic interstitial lung disease (ILD) belonging to the group of idiopathic interstitial pneumonias [1,2]. Its diagnosis requires the identification of a usual interstitial pneumonia (UIP) pattern in the appropriate clinical context after exclusion of other known causes of ILD [3,4]. High-resolution computed tomography (HRCT) plays a central role in the diagnosis of UIP, and multidisciplinary discussion (MDD) is essential when clinical, radiological, and pathological findings require integration. The 2018 ATS/ERS/JRS/ALAT guideline classified HRCT findings as UIP, probable UIP, indeterminate for UIP, or an alternative diagnosis and conditionally recommended SLB for patients with probable or indeterminate UIP when a confident diagnosis could not be established through MDD [3]. In clinical practice, however, the decision to perform surgical lung biopsy (SLB) remains challenging because some patients may not tolerate the procedure; SLB carries a risk of morbidity and mortality, and histopathological findings may not always change diagnosis or management. The 2022 ATS/ERS/JRS/ALAT update allows a probable UIP pattern to be managed similarly to UIP when the clinical context and MDD support IPF and there is no concern for an alternative diagnosis. In such cases, histological confirmation is generally unnecessary. An indeterminate UIP pattern, by contrast, still requires integration of clinical, radiological, and, when appropriate, pathological information [4].

The primary aim of this study was to determine how often SLB was performed in patients with probable or indeterminate UIP patterns and to compare the final diagnoses established during follow-up through MDD in patients who did and did not undergo biopsy before publication of the 2022 guideline. We also assessed biopsy findings, final diagnoses, treatment patterns, disease progression, and long-term survival. Because the 2022 guideline was published after the study period, we interpreted these historical real-world findings in the context of the updated diagnostic approach.

## 2. Methods

### 2.1. Study Design and Setting

**Figure 1:**
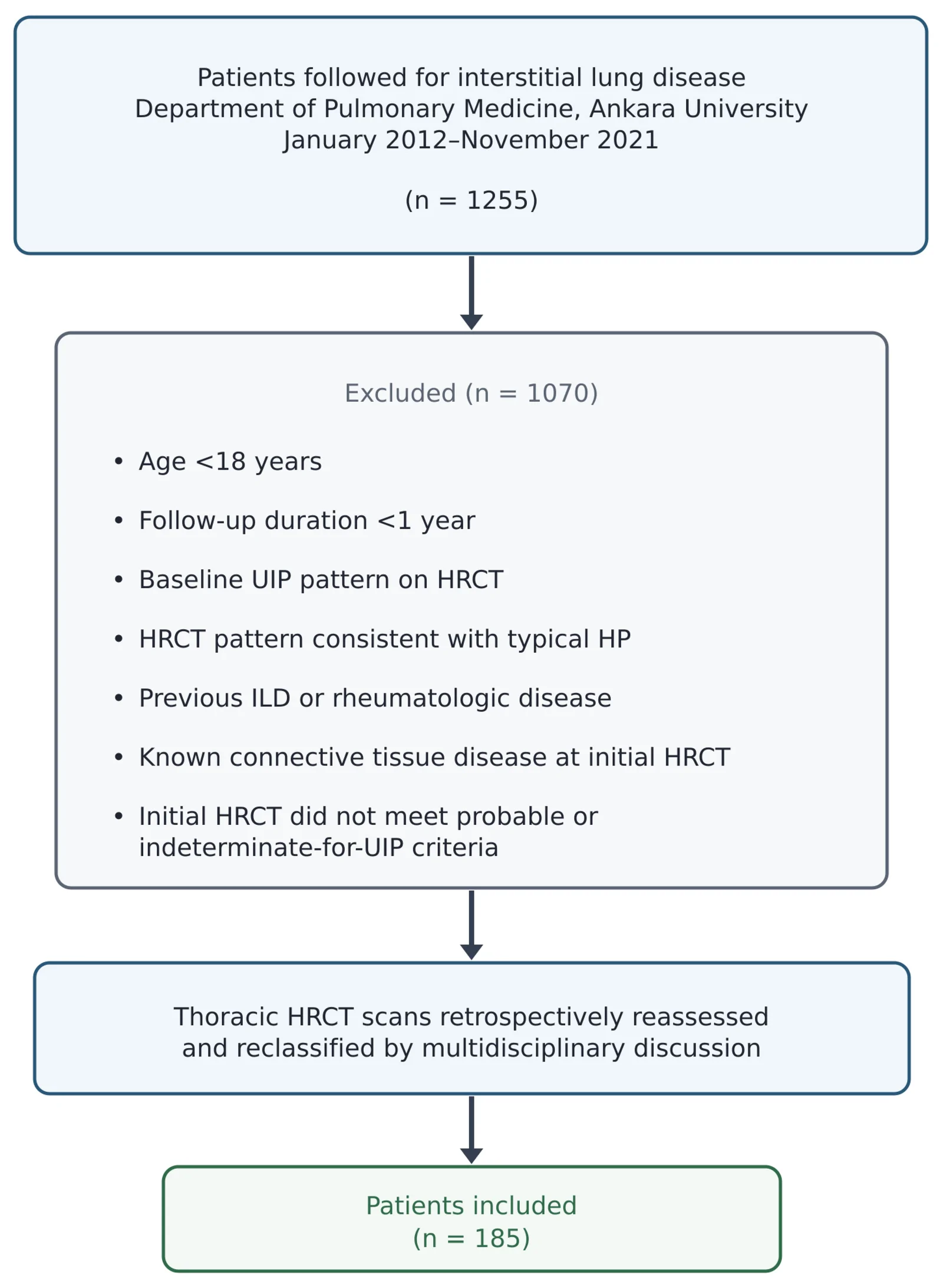
Flow diagram of patient selection. HRCT, high-resolution computed tomography; HP, hypersensitivity pneumonitis; ILD, interstitial lung disease; IPF, idiopathic pulmonary fibrosis; UIP, usual interstitial pneumonia.

In this retrospective study, thoracic HRCT scans of 1255 patients with an ILD diagnostic code who were followed between January 2012 and November 2021 at the Department of Pulmonary Medicine, Ankara University Faculty of Medicine, a tertiary referral center for ILD, were reviewed. Of these, 185 patients met the study criteria and were included in the analysis (Figure 1).

Ethical approval was obtained from the Ethics Committee of Ankara University Faculty of Medicine (Decision No: İ05-233-22, dated 13 June 2022).

Demographic, clinical, treatment, and outcome data were retrospectively extracted from the hospital’s electronic medical records.

### 2.2. Patient Selection

HRCT scans of patients aged 18 years or older were reviewed. All patients had been followed for at least one year and had no prior history of ILD and/or rheumatologic disease. We evaluated patients whose initial HRCT reports included terms such as probable UIP, indeterminate for UIP, interstitial fibrosis, or ILD, and included those who met criteria for a probable UIP or indeterminate for UIP pattern.

HRCT patterns were classified as probable UIP or indeterminate for UIP according to the 2018 ATS/ERS/JRS/ALAT criteria. These criteria were selected because the study cohort covered the period from 2012 to 2021 and the 2022 guideline had not yet been published. The 2022 update was therefore considered when interpreting the clinical implications of the findings but was not retrospectively applied to reclassify HRCT patterns or final diagnoses.

Patients were required to have at least 1 year of follow-up and a control HRCT scan. Patients with a baseline diagnosis of IPF, those whose initial HRCT was consistent with typical HP, and those diagnosed with CTD at the time of their initial HRCT were excluded.

No formal sample-size calculation was performed; all patients who met the eligibility criteria during the study period were included.

### 2.3. Multidisciplinary Assessment and Biopsy Strategy

The HRCT scans were re-evaluated and classified by a multidisciplinary discussion (MDD) comprising one thoracic radiologist, one pulmonary pathologist, one rheumatologist, and at least three pulmonologists specializing in ILD.

During the study period, the decision to offer SLB was individualized after clinical and multidisciplinary assessment rather than governed by a prospectively specified threshold. SLB was considered for patients without a definite UIP pattern on HRCT or an established diagnosis of HP or CTD, provided they were clinically fit and consented to the procedure. Procedural risk, expected diagnostic benefit, and patient preference informed the decision. For this retrospective analysis, clinical biopsy success was defined as a histopathological interstitial pattern that, after post-biopsy MDD, established or revised the final diagnosis and/or informed a treatment decision. A named pathological pattern alone was not counted as clinically successful if it did not contribute to the final MDD diagnosis or management. We report this clinical utility measure separately from histopathological diagnostic yield.

The distribution of final diagnoses was evaluated in patients who initially had a probable UIP or indeterminate for UIP pattern on baseline HRCT.

### 2.4. Definitions and Outcomes

Final diagnoses were determined through the initial MDD, serologic marker positivity during follow-up, surgical lung biopsy in selected cases, and subsequent MDDs during follow-up.

Disease progression was retrospectively defined as worsening respiratory symptoms documented during follow-up together with a qualitative increase in fibrotic abnormalities on serial HRCT examinations. Symptomatic worsening was determined from clinical records based on documented worsening of dyspnea, cough, or exercise tolerance. Radiological progression was assessed by comparing serial HRCT examinations for an increase in fibrotic abnormalities, including reticulation, traction bronchiectasis or bronchiolectasis, and honeycombing. Because of long-term follow-up, patients whose initial radiological pattern was indeterminate but later evolved into a UIP pattern were also considered to have progressive disease. Radiological progression was assessed qualitatively; no predefined quantitative or semiquantitative fibrosis scoring system was used. Pulmonary function data were not consistently available at standardized intervals and were therefore not incorporated into the progression definition.

To evaluate prognosis, data were recorded on post-diagnosis treatments, long-term oxygen therapy (LTOT), referral for lung transplantation, mortality, and overall survival.

### 2.5. Statistical Analysis

Data were analyzed using SPSS software package (Version 11.5, Chicago, IL, USA). Continuous variables were presented as mean ± standard deviation or median and range, as appropriate, and categorical variables were summarized as frequencies and percentages. Between-group comparisons were performed using Student’s *t*-test or the Mann–Whitney U test for continuous variables and Pearson’s chi-square or Fisher’s exact test for categorical variables, as appropriate.

Overall survival was calculated from the date of diagnosis to the date of death for patients who died and from the date of diagnosis to the last documented follow-up for patients who remained alive. Patients who were alive at the end of follow-up were censored at their last documented follow-up. Survival distributions were estimated using the Kaplan–Meier method and compared using the log-rank test. Univariable Cox proportional hazards regression analyses were performed to estimate the crude associations between the examined variables and all-cause mortality. A primary multivariable Cox regression model including baseline HRCT pattern, age at diagnosis, sex, and smoking history was used to estimate the adjusted association between HRCT pattern and all-cause mortality. Tied event times were handled using the Breslow method. The proportional hazards assumption was evaluated by adding interaction terms between each covariate and the natural logarithm of survival time to the multivariable model. Additional exploratory sensitivity analyses were conducted by adding antifibrotic treatment and cardiovascular disease to the primary model. Because antifibrotic treatment was initiated during follow-up, estimates from models including this variable were considered exploratory and were not interpreted as causal treatment effects. Results were reported as hazard ratios (HRs) with 95% confidence intervals (CIs). There were no missing data for the variables included in the Cox regression analyses. All tests were two-sided, and a *p*-value of <0.05 was considered statistically significant.

## 3. Results

### 3.1. Patient Characteristics

Overall, 185 patients were included in this study. The mean follow-up duration was 5.17 ± 3.35 years. The demographic and clinical characteristics of the study population are presented in Table 1.

### 3.2. Comparison by HRCT Pattern

Patients were classified radiologically as having either a probable UIP pattern or an indeterminate for UIP pattern. The demographic and clinical characteristics according to HRCT pattern are presented in Table 2.

### 3.3. Biopsy Use and Final Diagnosis

Among the 185 patients evaluated for diagnosis, 20 underwent surgical lung biopsy (SLB). SLB was performed in three patients with an indeterminate for UIP pattern and in 17 patients with a probable UIP pattern. SLB met predefined criteria for clinical biopsy success in 10 of 20 patients (50%). Among the 10 biopsies that did not meet the predefined criteria for clinical biopsy success, five patients remained classified as having unclassifiable ILD after SLB and subsequent MDD. In three patients, nonspecific histopathological findings did not contribute to the final diagnosis, which was established independently through MDD. In the other two patients, the histopathological findings did not provide additional diagnostic information or alter the treatment decision. Overall, two patients with unsuccessful biopsies were subsequently diagnosed with CTD-ILD during follow-up; these diagnoses were established independently of the biopsy findings. No SLB-related complications were documented. Detailed patient-level findings are presented in Supplementary Table S1.

Among the 10 patients in whom SLB met the predefined criteria for clinical biopsy success, nine (90%) had a probable UIP pattern on pre-biopsy HRCT. Among the 38 patients with a probable UIP pattern who were ultimately diagnosed with IPF, 32 (84.2%) received the diagnosis without SLB. The remaining six (15.8%) underwent SLB; in five of these patients, the biopsy met the predefined criteria for clinical biopsy success. In patients diagnosed with IPF who had an indeterminate UIP pattern, the diagnosis was clarified through MDD and longitudinal clinical and radiological follow-up without surgical biopsy. The final diagnoses were IPF in 23.8%, HP in 16.8%, CTD-ILD in 16.2%, fibrotic nonspecific interstitial pneumonia (fibrotic NSIP) in 14.1%, unclassifiable ILD in 11.4%, combined pulmonary fibrosis and emphysema (CPFE) in 7.6%, smoking-related ILD in 3.8%, interstitial pneumonia with autoimmune features (IPAF) in 3.2%, radiation- or drug-induced ILD in 0.5%, organizing pneumonia in 0.5%, eosinophilic lung disease in 0.5%, familial pulmonary fibrosis in 0.5%, sarcoidosis with CTD-ILD in 0.5%, and asbestosis in 0.5% (Table 3).

Among patients with a probable UIP pattern, the three most common final diagnoses were IPF in 26.4%, HP in 20.1%, and CTD-ILD in 17.4%. Among patients with an indeterminate for UIP pattern, the most frequent final diagnoses were unclassifiable ILD (26.8%), IPF (14.6%), and fibrotic NSIP (14.6%).

### 3.4. Treatment, Progression, and Survival

LTOT was initiated in 41.6% of the patients. Overall, 25.4% were followed without treatment, 42.7% received immunosuppressive therapy alone, 17.3% were treated only with antifibrotic agents, and 14.6% received combined immunosuppressive and antifibrotic therapy.

Antifibrotic treatment was initiated in 31.9% of the total cohort. When treatment distributions were compared according to HRCT patterns, no statistically significant difference was observed (*p* = 0.105).

Among patients followed in our department, 2.0% improved, 31.9% remained stable, and 65.9% showed disease progression. Of those managed without treatment, 53.2% remained stable, whereas 46.8% experienced progression. There was no statistically significant association between HRCT patterns and disease progression (*p* = 0.105).

Eighteen patients (9.7%) were considered eligible for lung transplantation and were referred; however, all died before transplantation could be performed.

**Figure 2:**
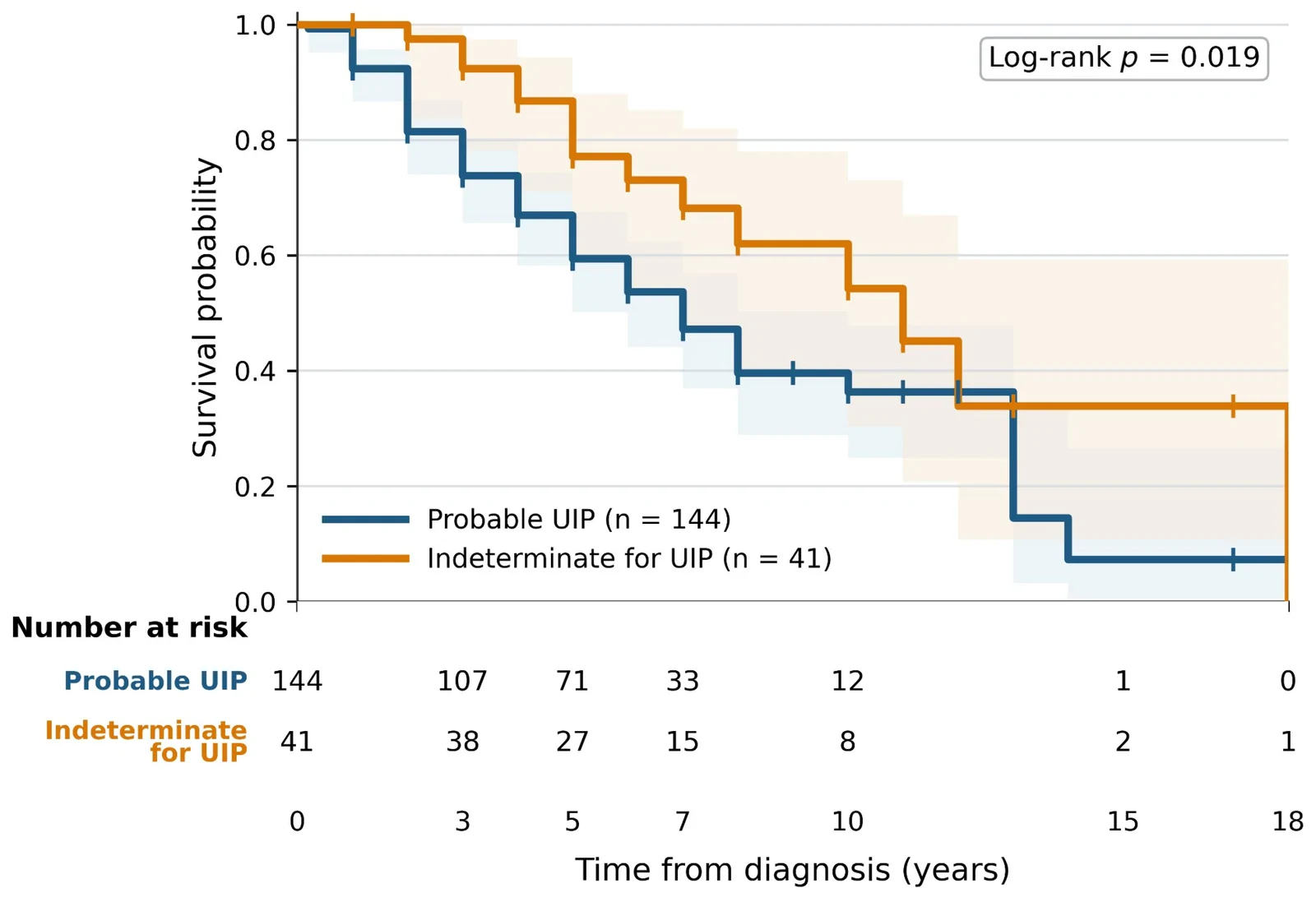
Kaplan–Meier survival according to baseline HRCT pattern (probable UIP vs. indeterminate for UIP). The shaded areas represent the 95% confidence intervals.

During follow-up, 85 of 185 patients (45.9%) died. Death occurred in 70 of 144 patients (48.6%) with a probable UIP pattern and in 15 of 41 patients (36.6%) with an indeterminate for UIP pattern. The difference in crude mortality proportions was not statistically significant (*p* = 0.173). The median overall survival of the entire cohort was 8 years. Median survival was 7 years in the probable UIP group and 11 years in the indeterminate for UIP group. The estimated 3- and 5-year survival rates were 73.8% and 59.4%, respectively, in the probable UIP group and 92.4% and 77.1%, respectively, in the indeterminate for UIP group. Kaplan–Meier analysis demonstrated significantly shorter survival in patients with a probable UIP pattern (log-rank *p* = 0.019) (Figure 2).

In univariable Cox regression analysis, a probable UIP pattern was associated with a higher mortality risk than an indeterminate for UIP pattern (HR 1.91, 95% CI 1.07–3.40; *p* = 0.028). In the primary multivariable model, adjusted for age at diagnosis, sex, and smoking history, probable UIP remained associated with a higher mortality hazard than indeterminate for UIP (adjusted HR 1.91, 95% CI 1.06–3.42; *p* = 0.030). Age, sex, and smoking history were not significantly associated with mortality in the multivariable model (Table 4).

No statistically significant time-dependent interactions were detected for baseline HRCT pattern, age, sex, or smoking history, and the global test did not indicate a violation of the proportional hazards assumption (Wald χ^2^ = 4.36, df = 4; *p* = 0.360).

In exploratory sensitivity analyses, the association between probable UIP pattern and mortality remained similar after additional adjustment for antifibrotic treatment (adjusted HR 1.90, 95% CI 1.05–3.42; *p* = 0.033). However, after additional adjustment for cardiovascular disease, the association was attenuated and was no longer statistically significant (adjusted HR 1.71,95% CI 0.93–3.14; *p* = 0.082). Cardiovascular disease was a composite variable encompassing hypertension, coronary artery disease, and heart failure. Of the 113 patients classified as having cardiovascular disease, 58 had hypertension without recorded coronary artery disease or heart failure; 21 of these 58 patients (36.2%) died, compared with 28 of the other 55 patients (50.9%). Thus, although probable UIP was associated with worse observed survival, whether this association was independent of relevant comorbidity remains uncertain. Results of all exploratory sensitivity models are presented in Supplementary Table S2.

## 4. Discussion

This real-world cohort yielded three principal findings. First, SLB was performed in only a small proportion of patients with probable or indeterminate UIP patterns and contributed to the final MDD diagnosis or treatment decision in half of those undergoing the procedure. Second, most patients with probable UIP who were ultimately diagnosed with IPF received the diagnosis through multidisciplinary and longitudinal assessment without SLB. Third, probable UIP was associated with shorter survival and a higher mortality hazard than indeterminate for UIP after adjustment for age, sex, and smoking history, although this association was attenuated after additional adjustment for cardiovascular disease.

In our study, SLB was performed in only a minority of patients and met the predefined criteria for clinical biopsy success in 10 of 20 cases (50%). Among patients with clinically successful SLB, nine (90%) had a probable UIP pattern on pre-biopsy HRCT. Among the 10 biopsies that did not meet the predefined criteria for clinical biopsy success, five patients remained classified as having unclassifiable ILD after biopsy and subsequent MDD. In three patients, nonspecific histopathological findings did not contribute to the final diagnosis, which was established independently through MDD. In the remaining two patients, the histopathological findings did not alter either the final diagnosis or treatment decision. Among the 38 patients with a probable UIP pattern who were ultimately diagnosed with IPF, six patients (15.8%) underwent SLB, and the biopsy met predefined criteria for clinical biopsy success in five of these six patients. The other 32 patients (84.2%) received the diagnosis without SLB. In addition, six patients with an indeterminate for UIP pattern were diagnosed with IPF based on longitudinal clinical and radiological assessment without undergoing SLB. Detailed patient-level findings are provided in Supplementary Table S1.

The clinical biopsy success rate in our cohort was lower than the approximately 80–95% histopathological diagnostic yield reported in previous SLB studies [5,6]. However, these outcomes are not directly equivalent because previous studies generally defined diagnostic yield according to the availability of a specific histopathological pattern, whereas our definition required the biopsy findings to contribute meaningfully to the final MDD diagnosis or treatment decision.

Although our SLB rate was lower than the approximately 20–34% reported in some IPF registries [7,8], it is consistent with the declining use of SLB in contemporary practice. For example, the UK IPF Registry reported a decrease in SLB use from 16.1% in 2013 to 2.8% in 2018 [8]. These comparisons should be interpreted cautiously because previous registries predominantly included patients with established IPF, whereas our cohort comprised patients with probable UIP or indeterminate for UIP patterns and encompassed heterogeneous final diagnoses.

The relatively low SLB rate may reflect clinician and patient preferences, advanced age, comorbidity burden, impaired pulmonary reserve, perceived procedural risk, and institutional practice. Although SLB can provide valuable diagnostic information, its potential benefits must be balanced against complications, including bleeding, pneumothorax, prolonged air leak, acute exacerbation, and perioperative mortality. These risks are greater in non-elective procedures and in patients with respiratory failure, severe physiological impairment, advanced age, or substantial comorbidity [5,6,9]. SLB is therefore most appropriate when the patient is an acceptable procedural candidate and histopathological findings are expected to meaningfully influence diagnosis or management. Such selective referral may have affected both the biopsy rate and diagnostic yield in our cohort.

The evolution from the 2018 to the 2022 ATS/ERS/JRS/ALAT diagnostic algorithm provides important context for our findings. However, because the cohort predated the publication of the 2022 guideline and HRCT patterns were classified according to the 2018 criteria, our study was not designed to directly evaluate the implementation or clinical impact of the 2022 guideline. Whereas the 2018 guideline conditionally recommended SLB for patients with probable or indeterminate UIP patterns, the 2022 update allows probable UIP to be managed similarly to UIP, with histological confirmation generally considered unnecessary when the clinical context supports IPF after MDD, and there is no concern for an alternative diagnosis [3,4]. In our cohort, 32 of 38 patients with probable UIP who were ultimately diagnosed with IPF received their diagnosis without SLB, while HP, CTD-ILD, and other ILDs were also common. Thus, probable UIP should not be equated with IPF without careful exclusion of alternative causes and MDD. For patients with indeterminate UIP, including six who were diagnosed with IPF through longitudinal assessment without SLB, our findings support continued integration of clinical, serological, and serial radiological information rather than diagnosis based on HRCT pattern alone. In clinical practice, the updated approach may reduce the need for SLB and facilitate earlier diagnostic and treatment decisions in appropriately selected patients with probable UIP, whereas patients with indeterminate UIP generally require further evaluation and longitudinal reassessment.

The broad distribution of final diagnoses in our cohort emphasizes that probable and indeterminate UIP are radiological patterns rather than disease-specific diagnoses. Although IPF was the most common final diagnosis (23.8%), HP (16.8%), CTD-ILD (16.2%), fibrotic NSIP (14.1%), and unclassifiable ILD (11.4%) together accounted for most cases. Previous studies have similarly demonstrated substantial diagnostic heterogeneity among patients with probable or indeterminate UIP patterns [10,11]. This heterogeneity has direct implications for clinical management because HRCT pattern alone should not determine the use of antifibrotic or immunosuppressive treatment. Initial MDD should integrate exposure history, autoimmune symptoms and serology, HRCT findings, and, when likely to alter management, bronchoscopic or surgical biopsy results. When diagnostic confidence remains insufficient, longitudinal reassessment incorporating the clinical course, serial pulmonary function tests, repeat HRCT, and reconsideration of exposure and autoimmune features may reveal diagnostic evolution. New or discordant findings should prompt repeat MDD and reconsideration of both diagnosis and treatment. This staged approach reserves tissue sampling for cases in which it is expected to provide clinically meaningful information [3,4].

Our findings provide real-world context for the 2022 guideline approach. Most patients with probable UIP who ultimately received a diagnosis of IPF did not undergo SLB. Across the overall study cohort, final diagnoses were largely established through MDD and longitudinal reassessment, with biopsy contributing in selected cases. Under the 2022 guideline, a probable UIP pattern may support an IPF diagnosis without histological confirmation when the clinical context and MDD provide sufficient confidence and there is no concern for an alternative diagnosis. Our experience illustrates the practical value of integrating clinical and radiological findings before deciding whether tissue sampling is likely to change management. MDD remains particularly important for patients with an indeterminate UIP pattern or features suggesting an alternative ILD. Because the cohort was assembled before publication of the 2022 guideline, our findings cannot establish its effect on biopsy use or patient outcomes.

Antifibrotic therapy was administered to 31.9% of the overall cohort. This proportion was lower than the rates of approximately 70% reported in contemporary IPF registries [12,13,14,15,16]. However, these rates are not directly comparable because our cohort included multiple ILD diagnoses, excluded patients with an established diagnosis of IPF at baseline, and covered a period during which antifibrotic availability and treatment practices changed. Among patients who received antifibrotic therapy, 62.7% had a final diagnosis of IPF, whereas the remaining patients were treated because of progressive fibrotic disease. Randomized trials have demonstrated that antifibrotic therapy can reduce the rate of lung-function decline in patients with IPF and in selected patients with progressive fibrosing ILDs [17,18,19,20,21,22,23]. Nevertheless, the present observational study was not designed to evaluate treatment effectiveness or the optimal timing of treatment initiation. Furthermore, because antifibrotic treatment was initiated during follow-up according to clinical status and disease progression, analyses involving treatment are susceptible to confounding by indication and time-dependent bias.

Overall, 65.9% of patients showed disease progression, whereas 31.9% remained stable, and no statistically significant difference in disease course or treatment distribution was observed between the probable and indeterminate UIP groups. These findings suggest that an indeterminate for UIP pattern should not necessarily be considered a benign or non-progressive radiological category. However, standardized longitudinal FVC and DLCO measurements were unavailable, and the study-specific definition of progression did not fully correspond to the subsequently published 2022 criteria for progressive pulmonary fibrosis. Therefore, patients treated for progressive fibrosis cannot be formally reclassified as having PPF on the basis of the available retrospective data. Our findings support careful longitudinal monitoring of both HRCT groups but do not allow conclusions regarding the benefit of early antifibrotic treatment.

Disease progression occurred in 69.4% of patients with a probable UIP pattern and 53.7% of those with an indeterminate for UIP pattern. Although progression was numerically more frequent in the probable UIP group, the difference did not reach statistical significance. Importantly, progression in more than half of the patients with an indeterminate pattern indicates that this radiological category should not be considered benign or clinically insignificant. Mura et al. similarly demonstrated that fibrotic progression may occur across different HRCT patterns in IPF: 8.9% of patients with probable UIP progressed to definite UIP, whereas 16.1% of those with an indeterminate pattern progressed to probable UIP [24]. These findings suggest that clinical and fibrotic progression may occur without radiological evolution to a definite UIP pattern. In selected patients who are ultimately diagnosed with IPF, an indeterminate pattern may represent an earlier radiological stage; however, this interpretation should not be generalized to all patients with an indeterminate pattern because this category includes several non-IPF ILDs. Taken together, our findings support careful longitudinal monitoring of both groups and demonstrate that an indeterminate for UIP pattern should not be interpreted as indicating a low risk of subsequent disease progression.

Survival differed according to baseline HRCT pattern. Although crude mortality proportions were not significantly different between the probable and indeterminate for UIP groups (70/144 [48.6%] vs. 15/41 [36.6%]; *p* = 0.173), Kaplan–Meier analysis demonstrated significantly shorter survival in patients with probable UIP. This apparent difference reflects the fact that Kaplan–Meier and Cox analyses account for follow-up duration and censoring, whereas crude mortality proportions do not. Median survival was 7 years in the probable UIP group and 11 years in the indeterminate for UIP group (log-rank *p* = 0.019). Probable UIP was associated with a higher mortality hazard after adjustment for age, sex, and smoking history (adjusted HR 1.91, 95% CI 1.06–3.42; *p* = 0.030). However, the association was attenuated and was no longer statistically significant after additional adjustment for cardiovascular disease (adjusted HR 1.71, 95% CI 0.93–3.14; *p* = 0.082). This change should be interpreted cautiously. Cardiovascular disease was more frequent in the indeterminate for UIP group, but the composite variable included patients with hypertension alone, among whom observed mortality was lower than in patients with other recorded cardiovascular conditions. This distribution may have contributed to the attenuation of the estimated association; it does not imply that hypertension protects against death. Differences in underlying ILD severity or clinical follow-up may also have influenced the result, but these explanations could not be tested adequately with the available retrospective data. The confidence interval indicates uncertainty regarding the magnitude of the association, particularly because the indeterminate for UIP group included only 41 patients and 15 deaths. Baseline HRCT pattern may provide prognostic information; however, residual confounding by comorbidity, underlying ILD diagnosis, and disease severity cannot be excluded, and the association requires confirmation in larger cohorts.

Importantly, the prognostic relevance of UIP morphology may not be confined to IPF. This consideration is particularly relevant to our diagnostically heterogeneous cohort, in which HP constituted 16.8% of the final diagnoses. In a recent multicenter study of fibrotic HP, a histopathological UIP-like pattern identified by transbronchial lung cryobiopsy was associated with greater functional decline and higher long-term mortality. Although histopathological and HRCT patterns are not directly interchangeable, these findings support the broader concept that UIP morphology may provide prognostic information across different fibrotic ILDs [25].

From a diagnostic perspective, transbronchial lung cryobiopsy (TBLC) has emerged as a less invasive method of obtaining histopathological samples in selected patients with undiagnosed ILD. Current international guidelines conditionally support TBLC as an acceptable alternative to SLB when performed and interpreted in centres with appropriate procedural and pathological expertise [4,26]. More recently, a randomized trial showed that a step-up strategy involving initial TBLC, with SLB reserved for cases in which TBLC was insufficiently informative, can reduce the need for SLB [27]. Nevertheless, careful patient selection, procedural standardisation, and multidisciplinary interpretation remain essential, and SLB may still be required after a non-diagnostic or clinically discordant TBLC.

Previous studies have similarly reported pattern-dependent differences in progression and mortality among patients with interstitial lung abnormalities and fibrotic ILDs [28,29,30,31]. Nevertheless, studies of interstitial lung abnormalities and cohorts restricted to IPF are not directly comparable with our clinically established and diagnostically heterogeneous ILD cohort. The estimated 3- and 5-year survival rates in our cohort were 78.0% and 63.4%, respectively, which were higher than the corresponding estimates reported in a meta-analysis of IPF cohorts (61.8% and 45.6%) [32]. This difference should be interpreted cautiously because our cohort included multiple ILD diagnoses and excluded patients with an established diagnosis of IPF at baseline. Overall, the results suggest that probable UIP is associated with less favorable survival than indeterminate for UIP, while the magnitude and independence of this association require confirmation in larger cohorts with standardized adjustment for physiological and radiological disease severity.

This study has several limitations. Its retrospective, single-center design and relatively small sample size limit the generalizability of our findings. In particular, the indeterminate for UIP group included only 41 patients and 15 deaths, limiting statistical power and the precision of adjusted estimates. Consequently, the confidence intervals were wide, particularly in the exploratory models that included additional covariates. The requirement for at least one year of follow-up and a follow-up HRCT may also have introduced selection bias. Standardized baseline and longitudinal pulmonary-function measurements, including FVC and DLCO, and quantitative assessments of radiological fibrosis extent were not consistently available. Therefore, adjustment for physiological and radiological disease severity was not possible. Quantitative CT may provide a more objective and reproducible assessment of fibrotic disease burden than visual evaluation; however, its measurements are influenced by CT dose, slice thickness, reconstruction parameters, respiratory status, and motion artifacts, which is particularly relevant in retrospective studies [33]. The absence of standardized assessments may have limited our ability to characterize disease severity and account for potential confounding effects. Future prospective multicenter studies should combine protocolized pulmonary function testing with harmonized HRCT acquisition and validated quantitative fibrosis scoring. The cohort also included patients with various final ILD diagnoses, each with distinct natural histories and prognoses, resulting in substantial clinical heterogeneity. Although adjustment for final ILD diagnosis or diagnosis-specific sensitivity analyses would have been informative, stratification by final diagnosis resulted in very small numbers of patients with an indeterminate UIP pattern within each diagnostic category, precluding a statistically reliable comparison with the probable UIP group. Residual confounding by disease severity, comorbidity, and underlying ILD diagnosis therefore cannot be excluded, and the observed association between HRCT pattern and survival should not be interpreted as a definitely established independent prognostic effect. Furthermore, the study-specific definition of progression did not fully correspond to the subsequently published 2022 criteria for progressive pulmonary fibrosis, and follow-up durations were recorded in years rather than months, limiting temporal precision. Nevertheless, the study provides long-term real-world data from a multidisciplinary ILD cohort with detailed longitudinal information on diagnosis, treatment, progression, and survival. The overall consistency of these real-world findings with the 2022 guideline framework further supports their clinical relevance.

## 5. Conclusions

In this real-world cohort, surgical lung biopsy was infrequently used in patients with probable or indeterminate UIP patterns, and most diagnoses were established through multidisciplinary and longitudinal assessment. Probable UIP was associated with shorter observed survival, and higher mortality hazard after adjustment for age, sex, and smoking history; however, the association was attenuated after additional adjustment for cardiovascular disease, leaving its independent prognostic significance uncertain.

Although outcomes were more favorable in patients with an indeterminate UIP pattern, frequent disease progression highlights the need for careful longitudinal monitoring of both groups. The use of multidisciplinary and longitudinal assessment to establish diagnoses in this cohort is consistent with the diagnostic flexibility introduced in the 2022 IPF guideline, which allows IPF to be diagnosed without biopsy in selected patients with probable UIP pattern following MDD. Biopsy decisions should nevertheless remain individualized, considering diagnostic uncertainty, procedural risk, and the likelihood that histological findings would change management. To further evaluate this diagnostic approach, prospective multicenter studies with standardized follow-up are needed to assess diagnostic evolution and long-term outcomes in patients managed without biopsy who lack both a definite UIP pattern and an established ILD diagnosis at baseline. Such studies could strengthen the generalizability of our findings and provide greater statistical power to compare outcomes across baseline HRCT patterns and subsequently established ILD diagnoses.

## Figures and Tables

**Table 1:** Baseline demographic and clinical characteristics of the study population.

Characteristic	Value
Patients, *n*	185
Age at Diagnosis, mean ± SD, years	60.83 ± 11.48
Female, *n* (%)	76 (41.1)
Male, *n* (%)	109 (58.9)
Comorbidities	
CVD, *n* (%)	113 (61.0)
DM, *n* (%)	52 (28.1)
GERD, *n* (%)	21 (11.4)
Malignancy, *n* (%)	14 (7.6)
COPD, *n* (%)	11 (5.9)
Smoking Exposure among ever-smokers, median (range) (pack years) ^b^	25 (0–100)
Smoking Exposure	
Never Smoker, *n* (%)	71 (38.4)
Former Smoker, *n* (%)	103 (55.7)
Current Smoker, *n* (%)	11 (5.9)
Exposure History	
Occupational Exposure, *n* (%)	72 (38.9)
Bird exposure, *n* (%)	57 (30.8)
Asbestos exposure, *n* (%)	55 (29.7)
Biomass, *n* (%)	53 (28.6)
Damp-environment exposure, *n* (%)	10 (5.4)
Hobby-related exposure, *n* (%)	8 (4.3)

CVD: Cardiovascular disease, COPD: Chronic obstructive pulmonary disease, DM: Diabetes mellitus, GERD: Gastroesophageal reflux disease. ^b^ Pack years were calculated among former and current smokers.

**Table 2:** Comparison of baseline demographic and clinical characteristics and mortality outcomes according to baseline HRCT pattern.

Characteristic	Probable UIP (*n* = 144)	Indeterminate for UIP (*n* = 41)	*p*-Value
Age at Diagnosis, mean± SD, years	61.99 ± 10.50	56.78 ± 13.81	0.010
Sex			
Female, *n* (%)	58 (40.3)	18 (43.9)	0.721
Male, *n* (%)	86 (59.7)	23 (56.1)	
Smoking exposure ever-smokers, median (range), pack years	30 (1–100)	20 (0–50)	0.629
Smoking Exposure			
Never Smoker, *n* (%)	55 (38.2)	16 (39.0)	
Former Smoker, *n* (%)	81 (56.3)	22 (53.7)	0.842
Current Smoker, *n* (%)	8 (5.6)	3 (7.3)	
Comorbidities			
CVD, *n* (%)	82 (56.9)	31 (75.6)	0.045
DM, *n* (%)	39 (27.1)	13 (31.7)	0.560
GERD, *n* (%)	17 (11.8)	4 (9.8)	1.000
COPD, *n* (%)	10 (6.9)	1 (2.4)	0.460
Malignancy, *n* (%)	9 (6.3)	5 (12.2)	0.198
Outcome			
Deaths, *n* (%)	70 (48.6)	15 (36.6)	0.173

CVD: Cardiovascular disease, COPD: Chronic obstructive pulmonary disease, DM: Diabetes mellitus, GERD: Gastroesophageal reflux disease.

**Table 3:** Distribution of final multidisciplinary diagnoses in the study cohort.

Final Diagnosis	N (%)
IPF	44 (23.8)
HP	31 (16.8)
CTD-ILD	30 (16.2)
Fibrotic NSIP	26 (14.1)
Unclassifiable ILD	21 (11.4)
CPFE	14 (7.6)
Smoking-related ILD (DIP and RB-ILD)	7 (3.8)
IPAF	6 (3.2)
Radiation- or drug-induced ILD	1 (0.5)
Organizing Pneumonia	1 (0.5)
Eosinophilic Lung Disease	1 (0.5)
Familial Pulmonary Fibrosis	1 (0.5)
Coexisting sarcoidosis + CTD-ILD	1 (0.5)
Asbestosis	1 (0.5)
Total	185 (100)

CPFE, Combined pulmonary fibrosis and emphysema; CTD-ILD, connective tissue disease-associated interstitial lung disease; DIP, Desquamative interstitial pneumonia; HP, Hypersensitivity pneumonitis; ILD: Interstitial lung disease; IPAF, Interstitial pneumonia with autoimmune features; IPF, Idiopathic pulmonary fibrosis; NSIP, Nonspecific interstitial pneumonia; RB-ILD, Respiratory bronchiolitis-associated interstitial lung disease.

**Table 4:** Univariable and multivariable Cox proportional hazards regression analyses of all-cause mortality.

Variable	Univariable HR (95% CI)	*p*-Value	Multivariable HR (95% CI)	*p*-Value
Probable UIP vs. indeterminate for UIP	1.91 (1.07–3.40)	0.028	1.91 (1.06–3.42)	0.030
Age at diagnosis, per 1-year increase	1.00 (0.98–1.02)	0.920	1.00 (0.98–1.02)	0.835
Female sex	0.85 (0.54–1.32)	0.465	0.83 (0.51–1.36)	0.457
Former or current smoking history	0.97 (0.63–1.51)	0.903	0.91 (0.56–1.48)	0.697

CI, confidence interval; HR, hazard ratio; UIP, usual interstitial pneumonia; Indeterminate for UIP, male sex, and never-smoking history were used as the reference categories. The analyses included 185 patients and 85 deaths. The multivariable model was adjusted for baseline HRCT pattern, age at diagnosis, sex, and smoking history.

## Data Availability

Data used and analyzed for this study are available from the corresponding author on reasonable request.

## Supplementary Materials

The following supporting information can be downloaded at: https://www.mdpi.com/article/10.3390/jcm15207786/s1, Table S1: Patient-level findings and clinical contribution of surgical lung biopsy; Table S2: Sensitivity analyses for the association between probable UIP pattern and mortality.

## Abbreviations

ALAT	Asociación Latinoamericana de Tórax
ATS	American Thoracic Society
COP	Cryptogenic organizing pneumonia
COPD	Chronic obstructive pulmonary disease
CT	Computed Tomography
CTD-ILD	Connective tissue disease-associated interstitial lung disease
DIP	Desquamative interstitial pneumonia
ERS	European Respiratory Society
HP	Hypersensitivity pneumonitis
HRCT	High-resolution computed tomography
ILD	Interstitial Lung Disease
IPAF	Interstitial pneumonia with autoimmune features
IPF	Idiopathic pulmonary fibrosis
JRS	Japanese Respiratory Society
LTOT	Long-term oxygen therapy
NSIP	Nonspecific interstitial pneumonia
PPF	Progressive Pulmonary Fibrosis
SLB	Surgical Lung Biopsy
UIP	Usual interstitial pneumonia
