# Supplementary material for: Real-World Management and Outcomes of Probable and Indeterminate UIP Patterns on HRCT: A Retrospective Cohort Study and Implications for the 2022 IPF Guideline
Source: J Clin Med. 2026 Oct 9;15(20):7786. doi: 10.3390/jcm15207786 (PMC13705359; doi:10.3390/jcm15207786)
Supplement: Supplementary file 1 [file jcm-15-07786-s001.zip › Supplementary Table S1.pdf]

Supplementary Table S1. Patient-level findings and clinical contribution of surgical lung biopsy.

Patient	Age/sex	HRCT pattern	Pre-biopsy diagnosis or differential diagnosis	Histopathological findings	Final MDD diagnosis	Clinical contribution of SLB	SLB success
1	48/M	Probable UIP	NSIP, DIP, or HP	Unclassifiable interstitial fibrosis	Unclassifiable ILD	No specific diagnosis; no effect on treatment	No
2	46/M	Probable UIP	HP	Subpleural fibrosis, DIP-like intra-alveolar macrophage accumulation, and focal honeycombing	Unclassifiable ILD	No specific diagnosis; no effect on treatment	No
3	55/M	Probable UIP	IPF	Nonspecific findings	CTD-ILD	Final diagnosis established during follow-up independently of SLB; no effect on treatment	No
4	52/F	Probable UIP	IPF	Honeycombing	IPF	Supported the final diagnosis and initiation of antifibrotic therapy	Yes
5	46/F	Indeterminate for UIP	NSIP	Consistent with NSIP	Fibrotic NSIP	Confirmed the final diagnosis; influenced treatment	Yes
6	56/M	Probable UIP	IPF	UIP	IPF	Confirmed the final diagnosis and led to initiation of antifibrotic therapy	Yes
7	39/M	Probable UIP	HP	Bronchocentric cellular interstitial pneumonia	HP	Supported and confirmed the final diagnosis; no additional effect on treatment	Yes
8	70/F	Probable UIP	HP or IPF	Nonspecific findings	HP	Final diagnosis established independently through MDD; no effect on treatment	No
9	54/F	Probable UIP	CTD-ILD or HP	Inflammatory cell infiltration with lymphoid follicles and aggregates, consistent with chronic HP	HP	Established the final diagnosis and changed treatment	Yes
10	47/F	Probable UIP	Fibrotic NSIP	UIP	Unclassifiable ILD	Clinicoradiological–pathological discordance prevented a definitive diagnosis; no effect on treatment	No
11	46/M	Probable UIP	HP	Consistent with UIP	IPF	Changed the diagnosis from HP to IPF and changed treatment	Yes
12	60/M	Probable UIP	Fibrotic NSIP	Upper-lobe-predominant UIP	HP	Contributed to changing the diagnosis from fibrotic NSIP to HP and changed treatment	Yes

Patient	Age/sex	HRCT pattern	Pre-biopsy diagnosis or differential diagnosis	Histopathological findings	Final MDD diagnosis	Clinical contribution of SLB	SLB success
13	54/M	Probable UIP	HP or fibrotic NSIP	Consistent with UIP	IPF	Established the final diagnosis and changed treatment	Yes
14	50/M	Probable UIP	Fibrotic HP or NSIP	Probable UIP	IPF	Established the final diagnosis and changed treatment	Yes
15	43/F	Probable UIP	Fibrotic NSIP	Nonspecific findings	HP	Final diagnosis established independently through MDD; no effect on treatment	No
16	44/F	Indeterminate for UIP	HP	Nonspecific findings	Unclassifiable ILD	No definitive diagnosis; no effect on treatment	No
17	25/M	Indeterminate for UIP	Sarcoidosis, PPFE, amyloidosis, or pulmonary alveolar microlithiasis	Nonspecific findings	Unclassifiable ILD	No definitive diagnosis; no effect on treatment	No
18	45/M	Probable UIP	NSIP, DIP, or UIP	Consistent with DIP	Smoking-related ILD	Established the final diagnosis and changed treatment	Yes
19	48/F	Probable UIP	Fibrotic NSIP or IPF	Probable UIP; considered nondiagnostic	CTD-ILD	Final diagnosis established during follow-up independently of SLB; no effect on treatment	No
20	58/M	Probable UIP	IPF	Probable UIP	IPF	Did not provide additional diagnostic certainty or change treatment	No

CTD-ILD, connective tissue disease-associated interstitial lung disease; DIP, desquamative interstitial pneumonia; HP, hypersensitivity pneumonitis; HRCT, high-resolution computed tomography; ILD, interstitial lung disease; IPF, idiopathic pulmonary fibrosis; MDD, multidisciplinary discussion; NSIP, nonspecific interstitial pneumonia; SLB, surgical lung biopsy; UIP, usual interstitial pneumonia.
