# Supplementary material for: Real-World Management and Outcomes of Probable and Indeterminate UIP Patterns on HRCT: A Retrospective Cohort Study and Implications for the 2022 IPF Guideline
Source: J Clin Med. 2026 Oct 9;15(20):7786. doi: 10.3390/jcm15207786 (PMC13705359; doi:10.3390/jcm15207786)
Supplement: Supplementary file 1 [file jcm-15-07786-s001.zip › Supplementary Table S2.pdf]

Table S2. Sensitivity analyses for the association between probable UIP pattern and mortality.

**Model**	**Adjustment Variables**	**HR (95% CI) for probable UIP**	***p*-value**
Model 1	Unadjusted	1.91 (1.07 – 3.40)	**0.028**
Model 2 (primary)	Age, sex, and smoking history	1.91 (1.06 – 3.42)	**0.030**
Model 3	Primary model + antifibrotic treatment	1.90 (1.05 – 3.42)	**0.033**
Model 4	Primary model + cardiovascular disease	1.71 (0.93 – 3.14)	0.082
Model 5	Primary model + antifibrotic treatment + cardiovascular disease	1.70 (0.93 – 3.14)	0.087

CI, confidence interval; HR, hazard ratio; UIP, usual interstitial pneumonia. Indeterminate for UIP was used as the reference category.
